# Escleroterapia de safena associada a enxerto de pele no tratamento de úlceras venosas

**DOI:** 10.1590/1677-5449.008217

**Published:** 2017

**Authors:** Alexandre Faraco de Oliveira, Horácio de Oliveira

**Affiliations:** 1 Universidade do Planalto Catarinense – UNIPLAC, Lages, SC, Brasil.; 2 Clínica Ana Carolina, Lages, SC, Brasil.

**Keywords:** úlcera de perna, úlcera varicosa, transplante de pele, escleroterapia, varizes

## Abstract

**Contexto:**

Úlceras são a resultante final de varizes associadas a refluxo de veias safenas.

**Objetivo:**

Demonstrar a possibilidade de associar dois procedimentos, a escleroterapia com espuma de veias safenas e o enxerto de pele parcial, para o tratamento de pacientes com úlceras venosas relacionadas a refluxo de veias safenas.

**Métodos:**

Foram tratados 20 membros em 20 pacientes, todos com ulcerações relacionadas a refluxo de veias safenas. Realizamos o enxerto de pele expandida, seguido da escleroterapia ecoguiada com espuma de polidocanol nas veias associadas às úlceras, através de punção ou dissecção da veia.

**Resultados:**

Em todos os casos, houve melhora dos sintomas relacionados à úlcera e cicatrização da lesão. Em 11 casos, obtivemos a viabilidade do enxerto de pele por completo; em quatro casos, houve cicatrização de cerca de 50% da lesão; e nos cinco casos restantes, houve cicatrização de aproximadamente 75% da lesão. A primeira ultrassonografia de controle revelou esclerose completa dos vasos tratados em 19 dos 20 casos e esclerose parcial sem refluxo detectável em um caso. Na segunda ultrassonografia, realizada após 45 dias, observamos esclerose completa de 15 casos; em cinco casos, houve esclerose parcial, dos quais três sem refluxo detectável e dois com refluxo em segmentos isolados associados a varizes. A complicação mais frequente foi a pigmentação nos trajetos venosos, observada em 13 pacientes. Um caso apresentou trombose assintomática de veias musculares da perna.

**Conclusão:**

Essa associação de procedimentos consiste em uma opção válida com potencial para promover um tratamento mais breve e de menor custo.

## INTRODUÇÃO

As úlceras de membros inferiores, relacionadas à doença venosa, costumam ser o estágio final de anos de tratamento inadequado ou da ausência de tratamento de um problema inicialmente simples e de fácil diagnóstico: as varizes de membros inferiores, em geral associadas a refluxo de veias safenas^1^. Com a úlcera instalada, o tratamento cirúrgico, que é frequentemente a solução definitiva para as varizes, tende a ser adiado até que as ulcerações estejam cicatrizadas para que a cirurgia possa ser realizada em melhores condições^1^.

Entretanto, as ulcerações relacionadas ao refluxo venoso podem ser bastante extensas e, de forma geral, exigem repouso prolongado com membros inferiores elevados por semanas ou meses, como forma de reduzir a pressão venosa associada à gênese da lesão e permitir a cicatrização. A aplicação do repouso como forma de tratamento, embora seja efetiva para cicatrização, tem frequentemente baixa adesão por parte dos pacientes, já descrentes de solução para suas lesões^1^^,^ 2.

Tendo em vista que a cicatrização da ulceração não determina a solução da doença, uma vez que sua causa está relacionada ao refluxo de veias varicosas, propomos um tratamento conjunto que proporcione a cobertura da lesão de pele, associado ao tratamento do refluxo venoso.

Neste trabalho, apresentamos uma série de casos em que utilizamos a associação de dois procedimentos realizados em sequência, na intenção de promover um tratamento mais rápido. Assim, pacientes que apresentavam úlceras de membros inferiores associadas a refluxo de veias safenas foram submetidos a esclerose com espuma dessas veias, seguida da cobertura das ulcerações com enxerto de pele parcial.

## METODOLOGIA

### Descrição dos casos

No período de janeiro de 2015 a dezembro de 2016, foram tratados 20 pacientes, todos com ulceras crônicas em membros inferiores de no mínimo 6 meses e no máximo 10 anos de evolução. Todas as cirurgias foram realizadas no mesmo hospital, e o trabalho foi aprovado pelo Comitê de Ética da instituição com protocolo nº 060617. Todos os pacientes apresentavam as veias safena magna, safena parva ou ambas com refluxo relacionado à área da ulceração.

Em todos os casos, foi utilizada espuma de polidocanol, obtida conforme a técnica de Tessari, com 1 mL de polidocanol e 4 mL de ar ambiente, produzindo 5 mL de espuma, sendo este procedimento repetido uma vez se necessário. Logo, para cada paciente, foram utilizados, no máximo, 10 mL de espuma. Utilizamos o polidocanol 3% para veias safenas e o polidocanol 1% para varizes colaterais. A aplicação da espuma foi realizada através de punção da veia com *scalp* ou através de sonda após dissecção de veia safena, dependendo do padrão de refluxo apresentado e da posição anatômica da úlcera. Todos os procedimentos foram realizados em ambiente cirúrgico, com paciente sob raquianestesia e em posição de Trendelenburg, com acompanhamento por ultrassonografia no momento da aplicação da espuma.

A estratégia adotada consistiu em realizar primeiramente a retirada da pele da área doadora utilizando um dermátomo. Na sequência, os segmentos de pele foram submetidos a um expansor, que promove um aumento de cerca de 50% da área através da realização de fenestrações nos segmentos de pele, seguido de limpeza da área receptora com lâmina de bisturi e cureta, implante da pele expandida fixada com pontos separados de *nylon*, punção da veia a ser tratada, preparo da espuma, esclerose do vaso, curativo e compressão com atadura de baixa elasticidade.

A área doadora de pele, em todos os casos, foi a coxa do mesmo membro em face anterolateral. Houve cuidado para deixar livre o trajeto da safena magna na coxa, de forma a permitir o acompanhamento da esclerose. O curativo primário foi uma placa de material altamente absorvente do tipo hidrofibra estéril impregnado com prata, que permaneceu de 15 a 20 dias. O curativo secundário foi trocado diariamente.

A área receptora recebeu como curativo primário uma tela de malha de algodão impregnado com parafina, que possui propriedades antiaderentes, de forma a reduzir a tração das áreas enxertadas na troca de curativos. Esses curativos foram trocados a cada 3 ou 4 dias dependendo da exsudação e da necessidade de realizar uma limpeza no leito da lesão. O curativo secundário foi trocado a cada 24 ou 12 horas conforme a exsudação da lesão.

Os pacientes foram orientados a permanecer a maior parte do tempo deitados com os membros elevados e a realizar caminhadas curtas de três a quatro vezes por dia. Realizamos uma primeira ultrassonografia de controle de 7 a 10 dias após a cirurgia e uma segunda ultrassonografia de 40 a 60 dias após a cirurgia.

## RESULTADOS

Foram tratados 20 membros inferiores em 20 pacientes, com idades entre 36 e 72 anos, sendo 14 mulheres e seis homens. As úlceras estavam associadas a refluxo concomitante de safena magna e parva em dois casos, de safena magna isolada em 13 casos e de safena parva isolada em cinco casos. Em quatro casos, existiam colaterais varicosas que também foram esclerosadas.

Observou-se melhora dos sintomas dolorosos em todos os casos, embora não tenhamos aplicado uma escala específica para medida da dor. Como a grande maioria dos pacientes (19 de 20) fazia uso prévio de medicação analgésica e anti-inflamatória antes da cirurgia, foi relatado como desnecessário o uso de qualquer medicação analgésica no retorno realizado após 45 dias. Todos os 10 pacientes que manifestavam prurido no período pré-operatório relataram redução ou ausência do sintoma no pós-operatório imediato e no retorno de 45 dias.

**Figura 1:**
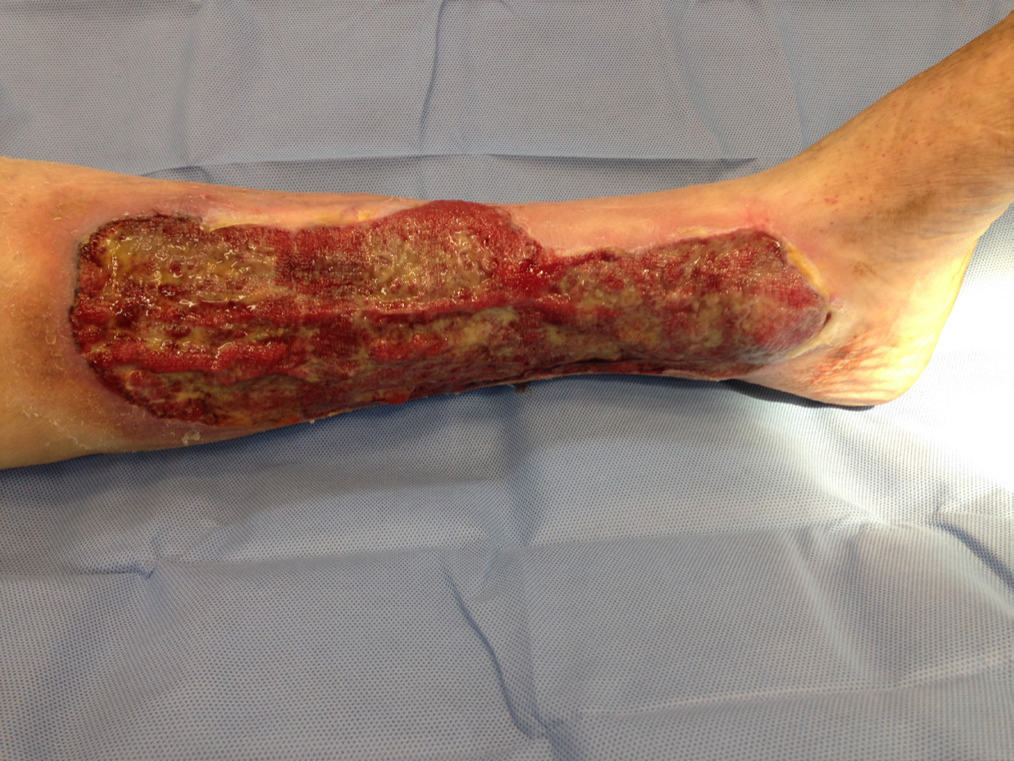
Úlcera antes da cirurgia.

**Figura 2:**
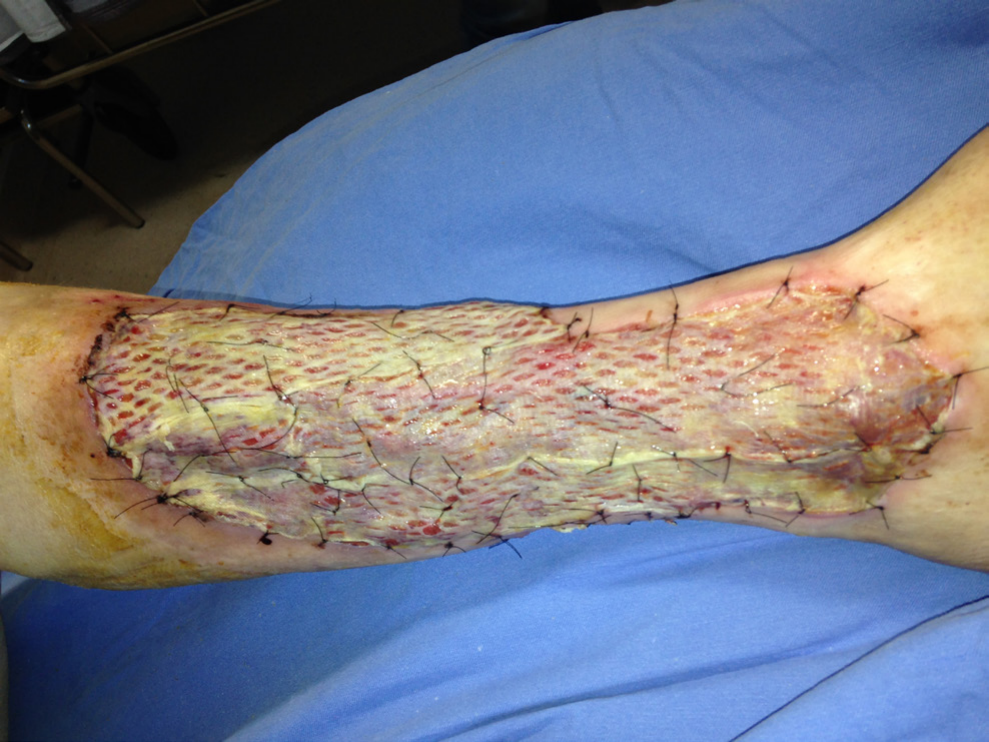
Úlcera com enxerto fixado por pontos, com 2 dias de pós-operatório.

Em 11 casos, obtivemos a viabilidade do enxerto de pele por completo, com cicatrização de toda a lesão. Em quatro casos, a cicatrização foi de cerca de 50% da lesão e, nos cinco casos restantes, foi de aproximadamente 75% da lesão (Figuras 1, 2 e 3).

**Figura 3:**
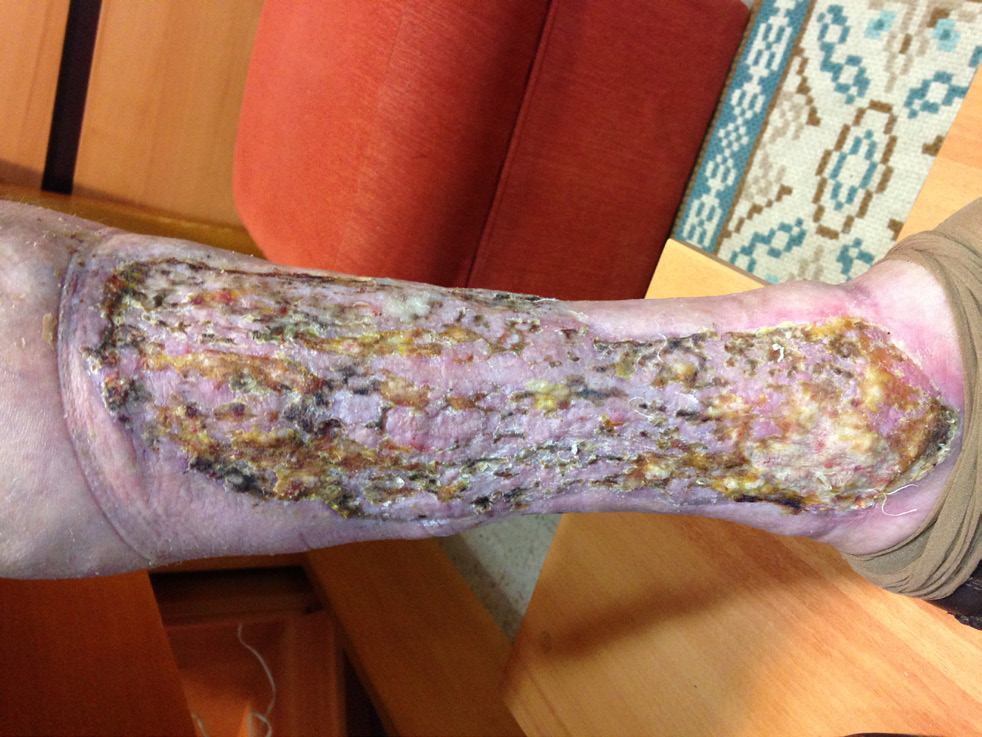
Úlcera com enxerto, com 60 dias de pós-operatório.

A primeira ultrassonografia de controle revelou esclerose completa dos vasos tratados em 19 dos 20 casos e esclerose parcial sem refluxo detectável em um caso. Na segunda ultrassonografia, realizada após 45 dias, observamos esclerose completa de 15 casos e esclerose parcial dos cinco casos restantes, dos quais três casos sem refluxo detectável e dois casos com segmentos isolados com refluxo associado a varizes.

A complicação mais frequente foi a pigmentação nos trajetos venosos, observada em 13 dos 20 pacientes. Em três casos, ocorreu tromboflebite em cerca de 50% do trajeto esclerosado, o que causou dor no local. Em outros cinco casos, observamos áreas de tromboflebite isoladas que não foram clinicamente significativas.

Não observamos trombose venosa profunda em nenhum dos casos na primeira ultrassonografia. Foi identificada trombose venosa assintomática de veias gastrocnêmias em uma paciente na segunda ultrassonografia. Nenhum paciente apresentou queixas visuais ou respiratórias significativas. Uma paciente apresentou tontura e hipotensão, com dispneia discreta no primeiro pós-operatório, e foi submetida a ecocardiografia e tomografia de tórax, que apresentaram resultados normais. Assim, a sintomatologia foi atribuída a uma resposta vagal.

## DISCUSSÃO

As varizes de membros inferiores são uma patologia bastante conhecida cujo quadro clínico costuma incluir inicialmente sintomas dolorosos em função do edema associado a estase nas veias varicosas. O último estágio da doença consiste na formação de lesões de pele e, finalmente, ulcerações, que podem ser bastante extensas e de difícil cicatrização. A úlcera relacionada à doença varicosa tende a ser mais frequente nas populações que não dispõem de uma assistência à saúde adequada, por ser resultado da falta de tratamento ou do tratamento inadequado das varizes^1^^,^ 3 ^,^ 4.

Outro fator que contribui para grandes ulcerações é o comportamento indolente das úlceras, que, embora não costumem apresentar dor intensa, provocam desconforto permanente. Esse desconforto normalmente é tolerável, e o alívio do sintoma costuma ser associado ao simples repouso com elevação dos membros inferiores. Dessa forma, muitos pacientes convivem com as úlceras venosas por meses e anos, utilizando múltiplas “receitas” de curativos para obter alívio parcial de suas queixas nos momentos de repouso e vivendo diversos episódios de exacerbação com a progressão das ulcerações em longo prazo^3^^,^ 5.

Para que tenhamos a cicatrização da ulceração, precisamos retirar a hipertensão venosa, relacionada ao refluxo das veias varicosas. Esse objetivo pode ser alcançado facilmente através de repouso com as pernas elevadas; entretanto, esse repouso precisa ser mantido de forma praticamente permanente por semanas ou meses, dependendo do tamanho da lesão, o que dificulta a adesão dos pacientes ao tratamento. Ainda que esses longos períodos de repouso sejam realizados, com cicatrização da ulceração, a causa subjacente que provocou a lesão ainda estará presente e, se não tratada, poderá promover a recidiva da lesão.

O tratamento da doença varicosa pode ser feito de forma clínica ou cirúrgica. O tratamento clínico costuma reduzir os sintomas e manter a doença sob controle com o uso diário de meias compressivas. O tratamento cirúrgico é aquele que consegue atuar sobre a causa das ulcerações e costuma ser o mais indicado para casos em que o refluxo e as varizes são importantes a ponto de causar ulcerações. Nos últimos anos, além da ressecção cirúrgica, diversas técnicas de ablação das veias safenas têm sido utilizadas para tratar o refluxo.

Existem duas abordagens para tratar as veias safenas sem retirá-las: utilizar algum tipo de dispositivo que realize a fototermólise da veia, como o *laser* ou a radiofrequência, ou injetar alguma substância esclerosante que produza a destruição do endotélio e subsequente fibrose local, como ocorre com as substâncias alcoólicas tensoativas, a exemplo do polidocanol^4^^,^ 6.

Entre as opções, a escleroterapia com espuma de grandes vasos é uma solução que permite alcançar veias onde o procedimento cirúrgico por vezes não é satisfatório, em virtude da intensa fibrose e do processo inflamatório subjacente. A esclerose de veias safenas, mesmo em áreas com ulcerações, pode ser conseguida com pouco traumatismo para o paciente e com custo reduzido na comparação com outros métodos como ablação térmica por *laser* ou radiofrequência^7^.

Existem diferentes evidências no que se refere à comparação dos métodos. Entretanto, em linhas gerais, há algum consenso de que a cirurgia tende a ser o método mais duradouro em longo prazo, apesar de costumar requerer bloqueio anestésico e gerar algum dano adicional aos tecidos fibrosados adjacentes às áreas com ulcerações. As técnicas que utilizam a ablação térmica não costumam requerer o bloqueio anestésico e tendem a ser menos traumáticas, porém possuem uma taxa de insucesso relacionada à recanalização e têm um alto custo relacionado aos equipamentos envolvidos^8^. A esclerose com espuma também é muito pouco traumática, dispensa anestesia e possui um percentual de recanalização que não é desprezível, porém tem a seu favor o baixo custo e a possibilidade de ser repetida sem prejuízo para o paciente^9^^-^ 11.

No que se refere a complicações, uma complicação grave costuma ser o tromboembolismo, que em tese estaria mais associado à escleroterapia com espuma do que às demais técnicas de tratamento de varizes. Entretanto, entende-se atualmente que se trata de um evento raro, menos de 1%^12^, e muito raramente fatal^13^. Embora ajam relatos de complicações extremamente incomuns, como infarto^14^, diversas séries que avaliaram complicações observaram uma frequência semelhante na comparação de diferentes técnicas^9^^,^ 12 ^,^ 15.

Após o tratamento do refluxo, ou seja, da causa da ulceração, restará a lesão ulcerada, que, ainda que esteja livre do agente causador, consiste em uma situação de difícil resolução devido aos danos crônicos existentes nos tecidos próximos à lesão. Existem diversas estratégias de curativos, desde a bota de Unna até os mais recentes, como a utilização de sistemas a vácuo. A aplicação de diferentes técnicas de enxerto de pele também podem ser adotadas^7^^,^ 16 ^-^ 19.

Neste trabalho, enxergamos a possibilidade de realizar uma abordagem simultânea e, portanto, com vantagens para o paciente, o médico e o sistema de saúde. Para o paciente, representa a oportunidade de tratar não apenas a causa da lesão, mas a própria ulceração, uma vez que a aplicação de um enxerto de pele promove, desde o primeiro dia de pós-operatório, uma redução na dor associada à lesão, sem a presença do curativo direto de parte da área lesada. Da mesma forma, a exsudação constante dessas lesões reduz progressivamente com o passar dos dias. Ainda que seja necessário um período prolongado de internação associada a repouso no leito, a partir da alta e principalmente após um período de cerca de 45 dias, esses pacientes podem retornar, no mínimo parcialmente e em muitos casos completamente, à sua rotina normal, com poucos cuidados ou sem que seja necessário qualquer cuidado adicional, exceto o uso da meia elástica.

Embora este não seja um estudo comparativo, pode-se supor que, para o sistema de saúde, a abordagem proposta será vantajosa no sentido de reduzir custos. Os pacientes que podem ser beneficiados com esses tratamentos costumam ter uma história de anos de evolução das úlceras, envolvendo a utilização de medicação oral e tópica em grandes quantidades, o custo de curativos, o afastamento de atividades laborais, o impacto na produtividade de outros membros da mesma família que prestam auxílio ao paciente, além de impactos psicológicos inerentes a doenças crônicas e recidivantes^7^^,^ 20 ^-^ 23.

Por fim, para o profissional médico, o tratamento desses pacientes pode parecer pouco interessante em múltiplos aspectos. A doença em si é bem conhecida e os meios que levam à resolução do problema estão disponíveis. Entretanto, o paciente que já passou por diversos médicos e se tornou descrente dos tratamentos propostos é um desafio adicional, por vezes de difícil abordagem. Tradicionalmente, os pacientes vitimados por essas ulcerações constituem populações de baixa renda e com atenção precária à saúde, o que, por si só, constitui um fator de risco para a existência dessas ulcerações. Logo, esses pacientes costumam se ver obrigados a utilizar a assistência pública de saúde com as limitações existentes no Brasil^21^^,^ 23 ^,^ 24.

## CONCLUSÃO

A utilização de escleroterapia com espuma para o tratamento de veias safenas com refluxo associadas a ulcerações de pele é de fácil realização e baixo custo, e apresenta bons resultados e poucas complicações. O enxerto de pele expandida para úlceras de estase venosa mostrou bons resultados e efetividade em promover a cicatrização da lesão.

As duas abordagens, enxerto e esclerose, podem ser realizadas durante um mesmo ato cirúrgico, em sequência, sem que haja prejuízo técnico ou limitações ao processo de recuperação subsequente. Tal proposta, podemos supor, promoverá um tratamento mais rápido no conjunto dos objetivos a serem alcançados e, portanto, reduzirá os custos necessários para esse tratamento.
