# Supplementary material for: Escleroterapia de safena associada a enxerto de pele no tratamento de úlceras venosas
Source: J Vasc Bras. 2017 Oct-Dec;16(4):270–5. [Article in Portuguese] doi: 10.1590/1677-5449.008217 (PMC5944303; doi:10.1590/1677-5449.008217)
Supplement: Translation

# Translation: Sclerotherapy of saphenous vein combined with skin grafting for treatment of venous ulcers

**Authors:** Alexandre Faraco de Oliveira, Horácio de Oliveira

**Affiliations:** 1 Universidade do Planalto Catarinense – UNIPLAC, Lages, SC, Brazil.; 2 Clínica Ana Carolina, Lages, SC, Brazil.

**Keywords:** leg ulcer, varicose ulcer, skin transplantation, sclerotherapy, varicose veins

## Abstract

**Background:**

Ulcers are the end result of varicose veins associated with reflux in saphenous veins.

**Objective:**

To demonstrate the possibility of combining two procedures, foam sclerotherapy of saphenous veins and skin grafting, to treat patients with venous ulcers related to reflux in saphenous vein.

**Methods:**

20 limbs were treated in 20 patients. All patients had ulcers related to saphenous vein reflux. We performed the grafting with expanded skin, followed by administration of ultrasound guided polidocanol foam sclerotherapy in veins associated with ulcers, accessed by puncture or dissection of the vein.

**Results:**

In all cases there was improvement of ulcer-related symptoms and healing of the lesion. In 11 cases we achieved full skin grafting viability. In four cases there was healing of about 50% of the lesion and in the other five cases approximately 75% of the lesion healed. The first control ultrasonographic examination revealed complete sclerosis of the vessels treated in 19 of 20 cases, with partial sclerosis in one case, but no detectable reflux. The second ultrasonographic examination performed at 45 days showed complete sclerosis in 15 cases. In five cases there was partial sclerosis, without detectable reflux in three and with reflux in isolated segments associated with varicose veins in two. The most common complication was pigmentation along vein paths, observed in 13 patients. In one case there was asymptomatic thrombosis of muscle veins of the leg.

**Conclusion:**

This combination of procedures is a valid option, with the potential to provide quicker and less expensive treatment.

## INTRODUCTION

Leg ulcers related to venous disease tend to be the final stage of years of inadequate treatment or of no treatment for a problem that is initially simple and easy to diagnose: varicose veins of the lower limbs, in general associated with reflux in the saphenous veins.^1^ Once an ulcer has formed, surgical treatment, which is frequently the definitive solution for varicose veins, tends to be delayed until ulcerations have healed to allow surgery to be performed under more favorable conditions.^1^

However, ulcerations related to venous reflux can be very extensive and, in general, demand prolonged rest with legs raised for weeks or months in order to reduce the venous pressure associated with formation of the lesion and allow healing to take place. Although prescribing rest is an effective form of treatment to enable healing, it is often met with poor compliance by patients who have come to believe that there is no solution for their ulcers.^1^^,^ 2

Bearing in mind that healing of the ulceration is not a solution for the disease, since its cause is related to varicose vein reflux, we propose a combined treatment that involves covering the skin lesion and treating the venous reflux.

In this article we present a series of cases in which we used a combination of two procedures conducted in sequence, with the intention of providing faster treatment. Patients with lower limb ulcers associated with reflux in saphenous veins underwent foam sclerosis of these veins followed by partial skin grafting to cover the ulcerations.

## METHODOLOGY

### Description of cases

From January 2015 to December 2016, a series of 20 patients were treated. All had chronic lower limb ulcers with onset a minimum of 6 months and a maximum of 10 years previously. All operations were performed at the same hospital and the research project was approved by the institutional Ethics Committee under protocol number 060617. All patients had reflux in great saphenous veins, small saphenous veins, or both, related to the area of ulceration.

In all cases, polidocanol foam was made using the "Tessari Technique" technique from 1 mL of polidocanol and 4 mL of room air to produce 5 mL of foam, and the procedure was repeated once if necessary. Thus, a maximum of 10 mL of foam was used per patient. We employed 3% polidocanol for saphenous veins and 1% polidocanol for collateral varicose veins. Foam was administered by vein puncture with a scalp vein set or via a catheter after dissection of the saphenous vein, depending on the reflux pattern and the anatomic position of the ulcer. All procedures were conducted in a surgical suite with the patient under spinal anesthesia and in the Trendelenburg position and were monitored using ultrasonography while the foam was administered.

The strategy employed began by harvesting skin from the donor area using a dermatome. Skin segments were then subjected to an expansion procedure, which increases area by around 50% by making fenestrations in the skin. The recipient area was then cleaned using a scalpel blade and a curette and the expanded skin was implanted and attached with separate nylon sutures. The vein to be treated was punctured, the foam was prepared, sclerosis of the vessel was performed, dressings were applied, and compression was applied with low elasticity bandages.

In all cases, the skin donor area was the anterolateral surface of the ipsilateral thigh. Care was taken to leave the path of the great saphenous vein in the thigh free, to enable monitoring of the sclerosis process. The primary dressing was a pad of highly-absorbent material - silver-impregnated sterile hydrofiber – and was left in place for 15 to 20 days. The secondary dressing was changed daily.

The primary dressing applied to the recipient area was paraffin cotton gauze, which has anti-adherent properties, in order to reduce traction on the grafted areas when dressings were changed. These dressings were changed every 3 or 4 days, depending on the quantity of exudate and the need to clean the wound bed. The secondary dressing was changed every 24 or 12 hours, as wound exudation required.

Patients were instructed to remain lying down the majority of the time with legs raised and to take three or four short walks per day. The first control ultrasonographic examination was performed 7 to 10 days after surgery and a second ultrasonographic examination was performed 40 to 60 days after surgery.

## RESULTS

A total of 20 lower limbs were treated in 20 patients, 14 women and six men with ages ranging from 36 to 72 years. Ulcers were associated with concomitant reflux of great and small saphenous veins in two cases, of the great saphenous vein only in 13 cases, and the small saphenous only in five cases. In four cases there were collateral varicose veins that were also treated with sclerosis.

The painful symptoms improved in all cases, although we did not administer a scale specifically for measuring pain. Sine the great majority of patients (19 out of 20) were already taking analgesic and anti-inflammatory medication before surgery, prescription of analgesic medication was recorded as unnecessary at the 45-day follow-up consultation. All of the 10 patients who had reported itching at preoperative consultations reported reduction or absence of this symptom in the immediate postoperative period and at 45-day follow-up.

In 11 cases we achieved full skin grafting viability and the lesions healed completely. In four cases there was healing of about 50% of the lesion and in the other five cases approximately 75% of the lesion healed (Figures 1, 2, and 3).

**Figure 1:** Ulcer before surgery.

**Figure 2:** Ulcer with graft attached by sutures, 2 days after the operation.

**Figure 3:** Ulcer with graft, 60 days after the operation.

The first control ultrasonographic examination revealed complete sclerosis of the vessels treated in 19 of 20 cases, with partial sclerosis and no detectable reflux in one case. The second ultrasonographic examination performed at 45 days showed complete sclerosis in 15 cases. In five cases there was partial sclerosis, without detectable reflux in three cases and with reflux in isolated segments associated with varicose veins in two.

The most common complication was skin pigmentation along vein paths, which was observed in 13 out of 20 patients. In three cases, there was thrombophlebitis of around 50% of the sclerosed segment, causing localized pain. In a further five cases, we observed isolated areas of thrombophlebitis that were not clinically significant.

We did not observe deep venous thrombosis in any patients at the first ultrasonography. Asymptomatic venous thrombosis was detected in the gastrocnemius veins in one patient at the second ultrasonography. None of the patients had significant visual or respiratory complaints. One patient suffered dizziness and hypotension, with discrete dyspnea in the initial postoperative period and was sent for echocardiography and a chest tomography, with normal results. These symptoms were therefore attributed to vagal response.

## DISCUSSION

Lower limb varicose veins are a very well-known pathology with initial clinical presentation that generally includes painful symptoms caused by edema associated with stasis in varicose veins. The last stage of the disease comprises formation of skin lesions and, finally, ulcerations, which can be very extensive and unlikely to heal. Ulcers related to varicose disease tend to be more common in populations who do not have access to adequate healthcare, since they are the result of failed or inadequate treatment of varicose veins.^1^^,^ 3 ^,^ 4

Another factor that contributes to large ulcerations is the indolent behavior of ulcers, which, although they don’t tend to cause intense pain, do provoke permanent discomfort. This discomfort is normally tolerable and relief from symptoms tends to be related to simple rest with lower limbs raised. As a result, many patients live with venous ulcers for months or years, using a variety of different “prescriptions” for dressings to achieve partial relief from their complaints when at rest and living through multiple episodes of exacerbation as ulcerations progress over the long term.^3^^,^ 5

In order to enable the ulceration to heal, we must relieve the venous hypertension, which is related to reflux from varicose veins. This objective can easily be achieved by remaining at rest with the legs raised; however, this position must be maintained practically constantly for weeks or months, depending of the size of the lesion, making it unlikely that patients will comply with the treatment. Even if these long periods of rest are observed, allowing the ulceration to heal, the underlying cause that provoked the lesion will still be present and, if left untreated, can cause the lesion to recur.

Varicose disease can be treated clinically or with surgery. Clinical treatment tends to reduce symptoms and keep the disease under control, by wearing compression stockings daily. However, surgical treatment that is capable of acting on the cause of the ulcerations and tends to be more indicated for cases in which the reflux and varicose veins are severe enough to cause ulcerations. Over recent years, in addition to surgical resection, many different techniques for saphenous vein ablation have been used to treat reflux.

There are two approaches for treating saphenous veins without removing them: the first employs some type of device to induce photothermolysis of veins, such as laser or radio frequency ablation, while the second is to inject a sclerosant substance that provokes destruction of the endothelium and subsequent localized fibrosis, as occurs with tensoactive alcoholic substances, such as polidocanol.^4^^,^ 6

One option, foam sclerotherapy of large vessels, is a solution that can be used to manage large veins where surgical procedures sometimes are unsatisfactory, because of the intense fibrosis and the underlying inflammatory process. Sclerosis of saphenous veins, even in areas with ulcerations, can be achieved causing little trauma to the patient and at a lower cost than other methods such as thermal ablation with laser or radio frequency.^7^

There is varying evidence from comparisons of methods. However, in general, there is a certain degree of consensus that surgery tends to be the most lasting method over the long term, although it requires anesthetic blockade and causes some additional damage to fibrotic tissues adjacent to areas with ulcerations. Techniques that employ thermal ablation do not tend to need anesthetic blockade and are generally less traumatic, but they have a failure rate that is related to recanalization and they involve higher cost, related to the equipment needed.^8^ Foam sclerosis is also untraumatic, does not need anesthesia, and is associated with a non-negligible percentage of recanalization, but factors in its favor are its low cost and the possibility of repeating the procedure without harming the patient.^9^^-^ 11

With regard to complications, one possible severe complication is thromboembolism, which in theory is more strongly associated with foam sclerotherapy than with the other techniques for treating varicose veins. However, it is now understood that this is a rare event, less than 1%,^12^ and is very rarely fatal.^13^ While there are reports of extremely uncommon complications such as myocardial infarction,^14^ several series that assessed complications have observed similar frequencies for the different techniques compared.^9^^,^ 12 ^,^ 15

After treating the reflux, i.e. the cause of ulceration, the ulcerous lesions will still remain, and even though the causative agent has been removed, they are very difficult to cure because of chronic damage to tissues close to the ulcer. There are many different dressing strategies, ranging from the Unna boot to more recent systems employing vacuum. A variety of different skin grafting techniques can also be employed.^7^^,^ 16 ^-^ 19

In this study, we see a possibility for taking a simultaneous approach that offers advantages for the patient, the physician, and the healthcare system. For the patient, it offers the option of treating not only the cause of the lesion, but the ulceration itself, since a skin graft reduces the pain associated with the lesion, from the first day after the operation, without the need for a direct dressing on the damaged area. Similarly, the constant exudation of these ulcers reduces progressively over the following days. Although it is necessary to admit the patient for a long period of bed rest, after discharge, and particularly after about 45 days, patients can return to their normal routines at least partially and very often completely, with few precautions or without the need for any additional precautions, except wearing elastic stockings.

Although this is not a comparative study, it can be assumed that the approach proposed here should be advantageous for the healthcare system in terms of cost reduction. Patients who could benefit from these treatments tend to have a history of years of ulcer progression, involving use of large quantities of oral and topical medications, the cost of dressings, time off work, impact on the productivity of other members of the same family who care for the patient, and also the psychological impacts of chronic and refractory diseases.^7^^,^ 20 ^-^ 23

Finally, for the physician, treatment of these patients may appear of little interest for several reasons. The disease itself is well-known and methods that can provide resolution of the problem are available. However, patients who have already seen many different physicians and no longer believe in the treatments that are proposed pose an additional challenge that is sometimes difficult to overcome. Traditionally, patients who are victims of these ulcerations are from low-income populations with poor healthcare, which is an independent risk factor for theses ulcerations. As a result, these patients tend to be obliged to use public healthcare services, with all the limitations that this implies in Brazil.^21^^,^ 23 ^,^ 24

## CONCLUSIONS

Use of foam sclerotherapy to treat saphenous veins with reflux associated with skin ulcerations is simple, low cost, and offers good results with few complications. Expanded skin grafts for venous stasis ulcers demonstrated good results and efficacy for lesion healing.

The two procedures, grafting and sclerosis, can be performed during a single operation, sequentially, with no technical compromise or limitations to the subsequent recovery process. We assume that this proposal will result in more rapid treatment to achieve the set of objectives and, therefore, reduce the expenditure needed for the treatment.
